# Intrinsic and extrinsic regulation of rhabdomyolysis susceptibility by Tango2

**DOI:** 10.1242/dmm.050092

**Published:** 2023-09-05

**Authors:** Euri S. Kim, Jennifer G. Casey, Brian S. Tao, Arian Mansur, Nishanthi Mathiyalagan, E. Diane Wallace, Brandie M. Ehrmann, Vandana A. Gupta

**Affiliations:** ^1^ Division of Genetics, Department of Medicine, Brigham and Women's Hospital Harvard Medical School, Boston, MA 02115, USA; ^2^ Department of Chemistry, University of North Carolina at Chapel Hill, Chapel Hill, NC 27599, USA

**Keywords:** Lipid homeostasis, Rhabdomyolysis, Skeletal muscle, Zebrafish

## Abstract

Rhabdomyolysis is a clinical emergency characterized by severe muscle damage, resulting in the release of intracellular muscle components, which leads to myoglobinuria and, in severe cases, acute kidney failure. Rhabdomyolysis is caused by genetic factors linked to increased disease susceptibility in response to extrinsic triggers. Recessive mutations in *TANGO2* result in episodic rhabdomyolysis, metabolic crises, encephalopathy and cardiac arrhythmia. The underlying mechanism contributing to disease onset in response to specific triggers remains unclear. To address these challenges, we created a zebrafish model of Tango2 deficiency. Here, we demonstrate that the loss of Tango2 in zebrafish results in growth defects, early lethality and increased susceptibility of skeletal muscle defects in response to extrinsic triggers, similar to TANGO2-deficient patients. Using lipidomics, we identified alterations in the glycerolipid pathway in *tango2* mutants, which is critical for membrane stability and energy balance. Therefore, these studies provide insight into key disease processes in Tango2 deficiency and have increased our understanding of the impacts of specific defects on predisposition to environmental triggers in TANGO2-related disorders.

## Teaser

**Summary:** A zebrafish model of Tango2 deficiency provides insights into key disease processes and the impacts of specific defects on predisposition to environmental triggers in rhabdomyolysis in *TANGO2*-related disorders.

## INTRODUCTION

Rhabdomyolysis is a complex medical disorder involving catastrophic failure of skeletal muscle homeostasis and integrity, resulting in muscle breakdown and release of muscle cytosolic content into the circulation (Cabrera-Serrano and Ravenscroft, 2022). Rhabdomyolysis can give rise to serious health complications such as myoglobinuria, cardiac arrhythmia and acute kidney injury. Clinical symptoms can include severe muscle weakness, myalgia and muscle swelling, with serum creatine kinase levels rising above 1000 U/l. Rare disease-causing mutations are associated with a small but significant subset (∼15%) of rhabdomyolysis patients. Environmental factors, such as viral infections (SARS-CoV-2, HIV), physical exertion and certain medications, are major contributing factors in combination with a genetic predisposition (Szugye, 2020; East et al., 1988; Rawson et al., 2017; Wu et al., 2022; van den Bersselaar et al., 2021; Knoblauch et al., 2013). Even in genetic forms of rhabdomyolysis, environmental factors increase the susceptibility to recurrent episodes of muscle breakdown (Kruijt et al., 2021). Environmental factors contributing to rhabdomyolysis have been mostly identified through life-threatening reactions to different triggers in the clinical population. A lack of clear understanding of the intrinsic disease mechanism also prevents investigation of the role that extrinsic factors have on increasing the susceptibility of muscle damage in predisposing genotypes.

Recessive mutations in *TANGO2* underlie a rare pediatric disorder resulting in encephalopathy, rhabdomyolysis and cardiac abnormalities (Lalani et al., 2016; Kremer et al., 2016; Miyake et al., 2022). *TANGO2* encodes the transport and Golgi organization 2 protein, first identified in a genetic screen for proteins required in constitutive protein secretion in *Drosophila* cells (Bard et al., 2006). Depletion of Tango2 results in the fusion of the endoplasmic reticulum (ER) and Golgi compartments in *Drosophila* cells. The fibroblasts of TANGO2-deficient patients exhibit a profound decrease in the ER area (Lalani et al., 2016). Functional studies in patient-derived fibroblasts have shown that TANGO2 is required for ER-Golgi trafficking in cells (Milev et al., 2021). Proteomic analysis of fibroblasts from TANGO2-deficient patients revealed significant changes in the components of the mitochondrial fatty acid oxidation pathway, the plasma membrane, the ER, the Golgi and the secretory pathway, indicating pleiotropic roles in the biology of the disease (Mingirulli et al., 2020). Some patients' fibroblasts also showed a defect in palmitate-dependent oxygen consumption, suggesting impaired mitochondrial fatty acid oxidation (Kremer et al., 2016; Heiman et al., 2022). In contrast, myoblasts from TANGO2-deficient patients exhibited no defects in mitochondrial structure and function but exhibited abnormalities in mitochondrial function under nutrient stress. This suggests that TANGO2 may have different functions in different cell types in response to extrinsic triggers (Bérat et al., 2021). These studies indicate that TANGO2 deficiency results in intrinsic metabolic defects exacerbated under stress conditions. However, a clear understanding of the disease processes resulting in the pathological state is still lacking.

*TANGO2* mutations result in clinical heterogeneity in affected patients. Muscle weakness and neurodevelopmental presentation often precede life-threatening complications of rhabdomyolysis, cardiac arrhythmias or cardiomyopathy. However, a clear genotype-phenotype correlation is lacking for these patients (Powell et al., 2021). The presence of variable phenotypes in different cell types suggests that TANGO2 may play diverse roles in various cell types *in vivo*. *Tango2* knockout mice exhibit normal development, lifespan and physiology (International Mouse Phenotyping Consortium, https://www.mousephenotype.org/data/genes/MGI:101825). Therefore, robust model systems are needed to understand the effect of TANGO2 deficiency on variable clinical presentation and to identify the pathological processes contributing to disease onset and progression. To address these challenges, we developed vertebrate zebrafish models of Tango2 deficiency. Tango2 deficiency resulted in normal embryonic development in zebrafish but caused increased lethality during the larval and juvenile stages. The *tango2* mutant larval zebrafish developed acute muscle dysfunction by extrinsic stress triggers. Global lipidomics identified a reduced abundance of lipids synthesized by ER/sarcoplasmic reticulum (SR)-localized glycerol-3-phosphate pathway enzymes in Tango2 deficiency, which are critical for cellular membranes and energy states. The studies presented in this work provide mechanistic insights into intrinsic disease processes and extrinsic risk factors for increased susceptibility to rhabdomyolysis in *TANGO2*-related disorders.

## RESULTS

### Tango2 deficiency in zebrafish results in normal embryonic development but increased lethality during larval and juvenile stages

**Fig. 1.**
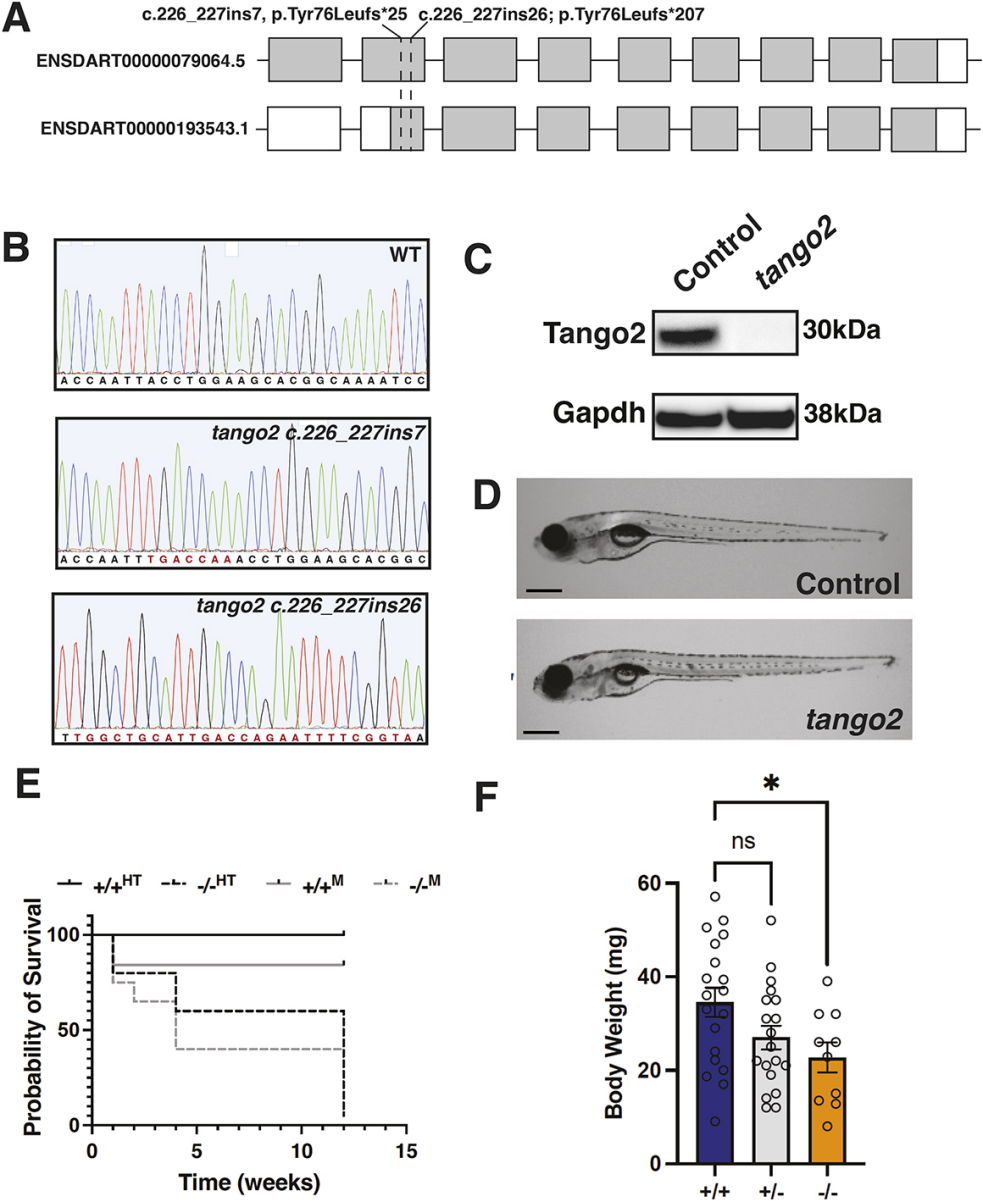
**Tango2 deficiency results in growth defects and lethality.** (A) Schematics depicting the position of *tango2* alleles (*tango2*^bwg210^ and *tango2*^bwg211^) with reference to both transcripts in zebrafish. *tango2* mutations resulted in insertions and frameshift mutations in different alleles. (B) Sanger sequencing for control and *tango2* alleles depicting mutations in *tango2*^bwg210^ (c.226_227ins7) and *tango2*^bwg211^ (c.226_227ins26). (C) Western blot analysis of proteins from control and *tango2* mutant fish (1 month of age). (D) Lateral view of control and *tango2* mutant larval zebrafish showing no phenotypic differences. Images are representative of *n*=6 larval fish. Scale bar: 0.5 mm. (E) Kaplan–Meier survival curve of control (+/+^HT^) and *tango2* mutants (−/−^HT^) obtained from heterozygous parents, and control (+/+^M^) and *tango2* mutants (−/−^M^) obtained from wild-type and mutant parents, respectively. *n*=360. (F) Body weight analysis of control (+/+ and +/−) and *tango2* mutants (−/−) at 1 month. *n*=60. Data are presented as mean±s.e.m. Unpaired two-tailed *t*-test, parametric; ns, not significant; *P<0.05.

Zebrafish grow *ex vivo*, so disease onset and progression can be visualized in individual animals. To understand the role of Tango2 *in vivo* in disease onset and progression, we created loss-of-function *tango2* alleles in zebrafish using the CRISPR-Cas9 system. The *tango2* gene in zebrafish encodes two transcripts, and therefore single guide RNAs (sgRNAs) were designed to knock out both transcripts (Fig. 1A). The *tango2* alleles created included *tango2*^bwg210^, with insertion of seven bases (c.226_227ins7; p.Tyr76Leufs*25), and *tango2*^bwg211^, with insertion of 26 bases in exon 2 (c.226_227ins26; p.Tyr76Leufs*207) (Fig. 1B). These alleles result in out-of-frame mutations and are predicted to result in truncated proteins. Phenotypic analysis of *tango2*^bwg210^ and *tango2*^bwg211^ lines revealed no significant differences. Therefore, the *tango2*^bwg211^ line (referred to as *tango2* mutants hereafter) was used for further investigation, unless otherwise specified. To validate the effect of the c.226_227ins26 mutation at the protein level, western blot analysis was performed on control and *tango2* zebrafish (4 weeks of age) and showed a complete absence of the Tango2 protein in the mutant fish (Fig. 1C; Fig. S1). Therefore, the c.226_227ins26 mutation results in the loss of function of the Tango2 protein in zebrafish. Phenotypic analysis of control and *tango2* mutant larval zebrafish showed no significant morphological differences during early development [Fig. 1D, 8 days post fertilization (dpf)]. To identify the effect of Tango2 deficiency on the lifespan of mutant fish, control (+/+^HT^) and mutant (−/−^HT^) genotypes obtained from heterozygous (‘HT’) parents were analyzed until 3 months of age. Despite no obvious morphological differences in control and mutant larval fish at early stages, a reduced survival rate was observed for mutant fish from 7 days post fertilization, and 96% of the mutant fish died by 3 months of age (Fig. 1E). *tango2* mutants (−/−^M^) obtained from *tango2* (mutant, ‘M’) parents also showed similar lifespans compared to those of *tango2* mutants (−/−^HT^) obtained from heterozygous parents, suggesting the effect of a lack of maternal *tango2* mRNA, reported previously for another allele of *tango2* (Sun et al., 2022). This was further validated by the absence of *tango2* mRNA in *tango2* mutants (−/−^HT^) (Fig. S1). Therefore, control and mutants obtained from the heterozygous *tango2*^bwg211^ line were used in subsequent studies. Quantifying body weight also showed a significant reduction in the weights of *tango2* mutants compared to those of wild-type siblings at 4 weeks of age, indicative of growth defects (Fig. 1F). Histological analysis of the skeletal muscle revealed a reduced myofiber size than in controls (Fig. S2). Taken together, these studies suggest that Tango2 deficiency in zebrafish results in muscle growth defects and early lethality in larval and juvenile stages.

### Tango2 is localized at endomembranes in myofibers

**Fig. 2.**
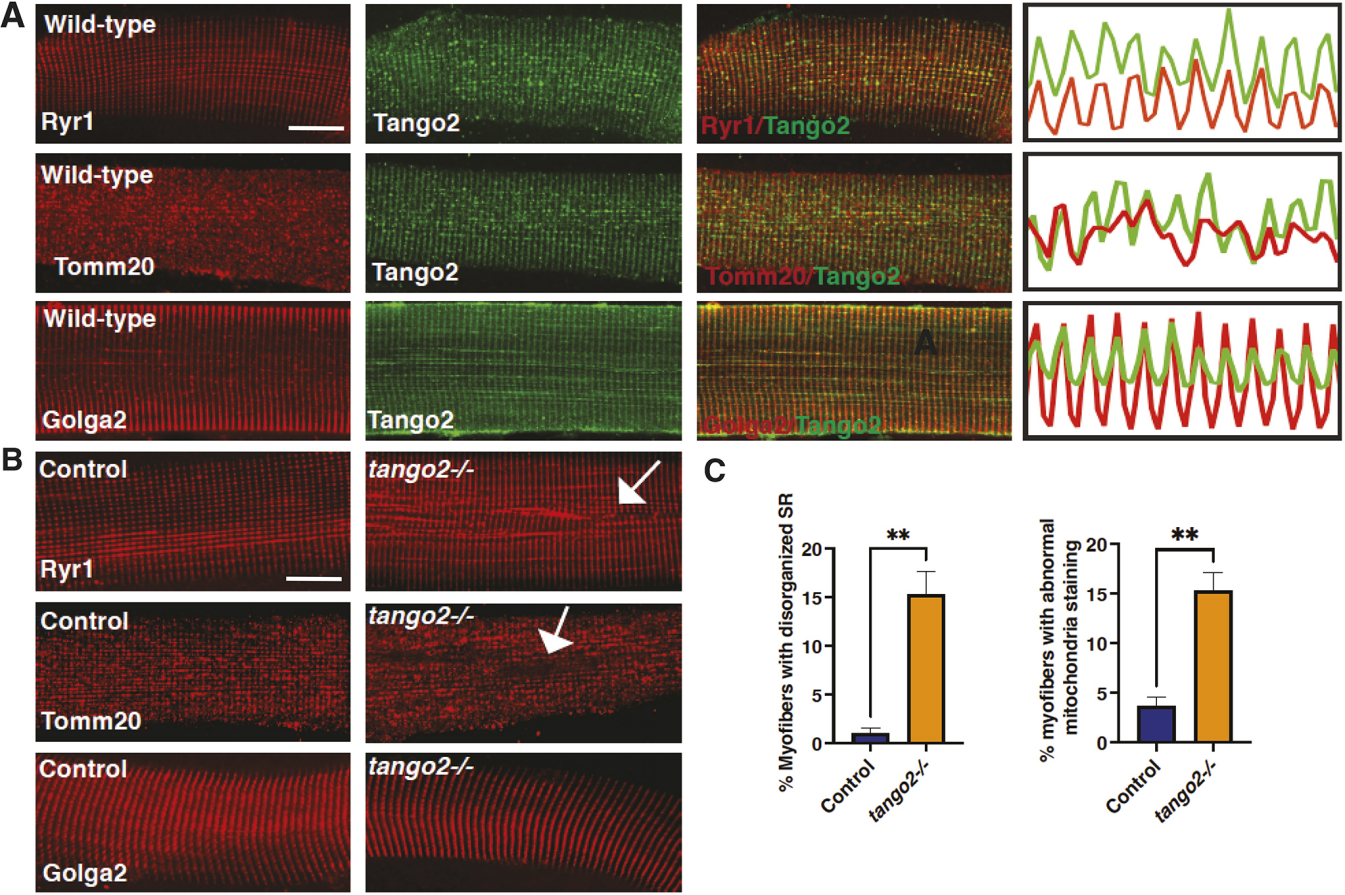
**Tango2 is required for the structural organization of SR and mitochondria.** (A) Myofibers were isolated from control (8-week-old) zebrafish and immunofluorescence for the SR (Ryr1), mitochondria (Tomm20) and the Golgi (Golga2) was performed. Single-channel densitometry tracing analysis of the longitudinal spatial profile was performed with colors corresponding to the staining pattern on the left, which showed that Tango2 localized with Ryr1 and Golga2. (B) Myofibers were isolated from control (8-week-old) and *tango2* mutant zebrafish and immunofluorescence was performed. *tango2^−/−^* mutant myofibers showed areas with disorganized or reduced immunoreactivity for Ryr1 and Tomm20 antibodies (white arrows). Myofibers were isolated from three different zebrafish for each genotype, and 10-15 myofibers were analyzed by plot profile in ImageJ in each group. Scale bars: 10 µm. (C) Quantification of the myofibers with abnormal areas with reduced or no staining for Ryr1 and Tomm20 in control and *tango2^−/−^* zebrafish. Data represent mean±s.e.m. from three different samples and 45-65 myofibers. **P<0.01, unpaired two-tailed Student's *t*-test.

*TANGO2* mutations result in a decreased network of the ER in patient fibroblasts and metabolic abnormalities, indicative of the involvement of intracellular organelles in disease pathology (Milev et al., 2021). To address the localization of Tango2 in skeletal muscle, immunofluorescence was performed on myofibers isolated from zebrafish (45 days of age). Colocalization with different skeletal muscle proteins showed colocalization of Tango2 with Ryr1 (collectively referring to the SR proteins Ryr1a and Ryr1b) and Golga2 (a Golgi apparatus protein) (Fig. 2A) in proximity to the mitochondria (Tomm20, collectively referring to Tomm20a and Tomm20b). Tango2 was previously identified in a genomic screen for the regulators of ER-Golgi trafficking. Therefore, the colocalization of Tango2 with the endomembrane compartments suggests potential roles in the maintenance or function of these organelles in the skeletal muscle (Bard et al., 2006). To understand the effect of Tango2 deficiency on these membrane compartments in skeletal muscle, immunofluorescence was performed on control and *tango2* mutant myofibers. Mutant myofibers displayed local regions of disorganized Ryr1 immunofluorescence, indicating structural defects in the SR in these areas (Fig. 2B, arrow in top-right panel; Fig. 2C). Quantification of the myofibers with regions of disorganized Ryr1 staining showed an increase in these regions in *tango2* mutant zebrafish (Fig. 2C). Moreover, regions with reduced or no mitochondrial staining were also observed in the mutant myofibers (Fig. 2B, arrow in middle-right panel; Fig. 2C). Quantification of these regions showed an increase in *tango2* myofibers compared to that in controls (Fig. 2C). No differences in the Golgi apparatus were identified between control and *tango2* mutant myofibers. Therefore, Tango2 is localized and required to maintain the structural organization of the endomembrane compartments.

### *tango2* mutant fish exhibit ultrastructural defects in the SR and mitochondria

**Fig. 3.**
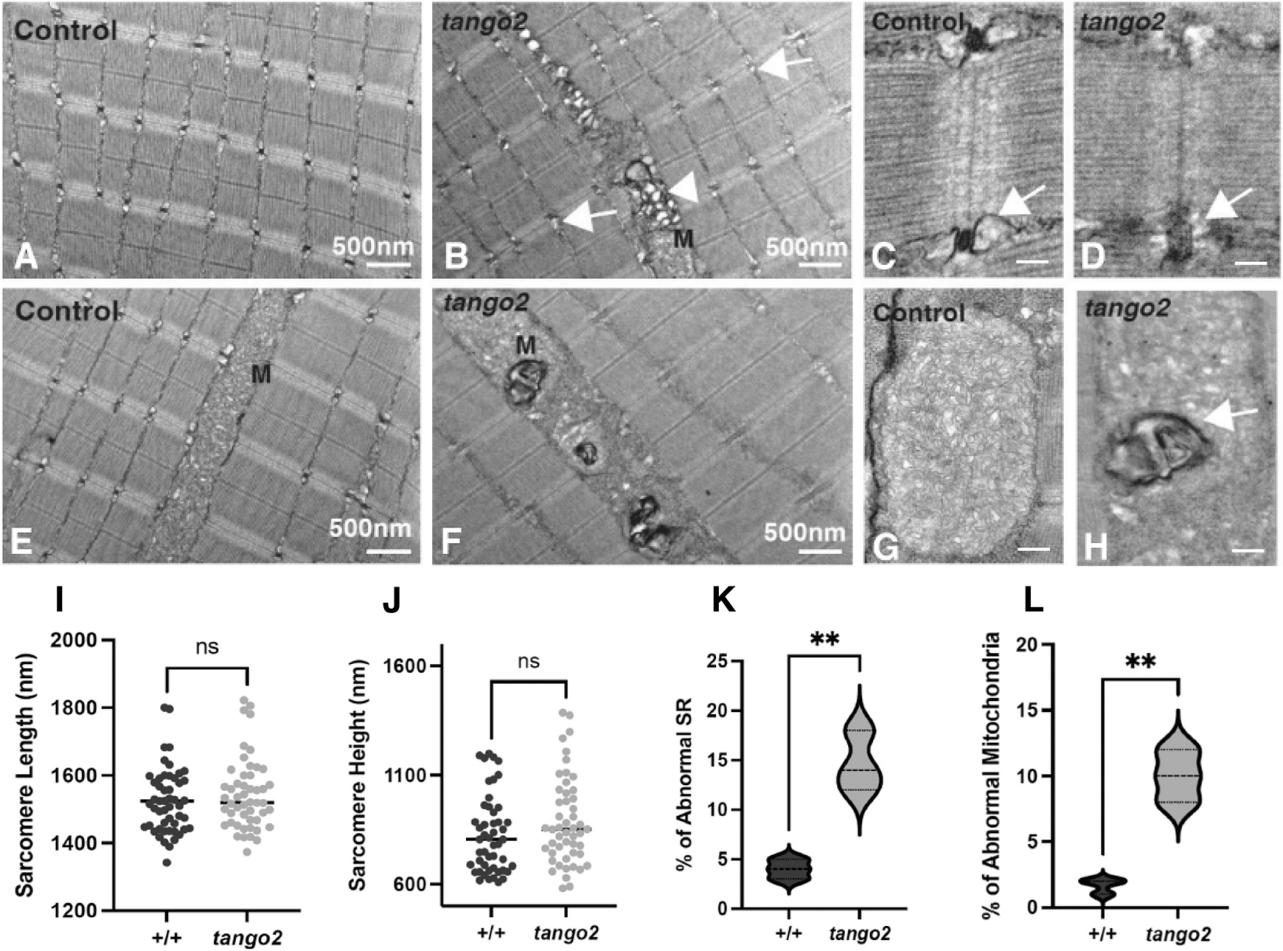
**Tango2 deficiency results in abnormal SR and mitochondria in skeletal muscle.** (A-H) Transmission electron microscopy images of the longitudinal sections of control and *tango2* mutant larval fish (8 dpf). (A,B,I,J) No abnormalities were present in the sarcomeres in *tango2* mutants (B) compared to controls (A). The SR in *tango2* mutants exhibited small or collapsed SR (arrows, B). The *tango2* mutant muscle also showed the presence of abnormal vesicular structures in the proximity of mitochondria (‘M’) and SR (white arrowhead, B). Quantification of the sarcomere length (I) and width (J) in control (+/+) and *tango2* mutant sarcomeres showed no significant differences. (C,D,K) The enlarged panels show normal SR (arrow) in controls (C) on either side of the darkly stained t-tubule. In comparison, *tango2* mutant SR (D) appears smaller or collapsed (arrow). Quantification of the abnormal SR (K) showed a significant increase in *tango2* mutants compared to controls. (E,F) Mitochondria in the control muscle (E) exhibited normal organization of the inner and outer mitochondrial membranes. A number of myofibers in *tango2* mutants (F) contained abnormal mitochondria with electron-dense whorled membrane structures. (G,H,L) Enlarged view of mitochondria in control myofibers (G) and *tango2* mutant myofibers (H), which showed abnormal mitochondria with electron-dense whorled membrane structures (arrow, H). Quantification of the mitochondria (L) showed an increase in mitochondria with whorled membrane structures in the *tango2* mutants. Data represent mean±s.e.m. from three different samples and 45-65 myofibers. ns, not significant; **P<0.01; two-tailed unpaired Student's *t*-test. Scale bars: 500 nm (A,B,E,F); 100 nm (C,D,G,H).

To identify the temporal changes of structural abnormalities observed in myofibers in larval fish (60 days of age, Fig. 2), skeletal muscle ultrastructure was examined during early larval development (8 dpf) when control and mutant fish are phenotypically and functionally similar. Ultrastructural evaluation of sarcomeres by electron microscopy showed no significant defects in sarcomere size (length or height) of *tango* mutants compared to that of control siblings (Fig. 3A,B,I,J). Although most of the SRs and mitochondria were normal in *tango2* mutants, 10-12% myofibers exhibited defects in both SR (13.8±4.9% in mutants versus 4.1±0.85% in control, indicated as mean±s.e.m.; *P*<0.01) and mitochondrial (9.2±2.3% in mutants versus 0.8%±0.79 in control, *P*<0.01) structures (Fig. 3K,L). Defective SR showed either collapsed or smaller terminal cisternae in *tango2* mutants compared to those in controls (Fig. 3B-D, arrows). In contrast to longer mitochondria in control muscles, *tango2* mutant muscles exhibited smaller mitochondria associated with whorled membrane structures (Fig. 3E-H, indicated by ‘M’), also seen in mitochondrial myopathies (Vincent et al., 2016). Interestingly, these abnormalities were present together in these affected myofibers, and no myofibers with either SR or mitochondrial defects were observed. Moreover, an abnormal accumulation of vesicles was also observed in the proximity of the SR and mitochondria that exhibited similar electron-dense membranes as whorled membrane structures in the mitochondria in the mutant myofibers (Fig. 3B, arrowhead), and were likely derived from damaged mitochondria. These data show that the absence of Tango2 results in ultrastructural defects in the SR and mitochondria.

### SR stress results in skeletal muscle defects in *tango2* mutants

**Fig. 4.**
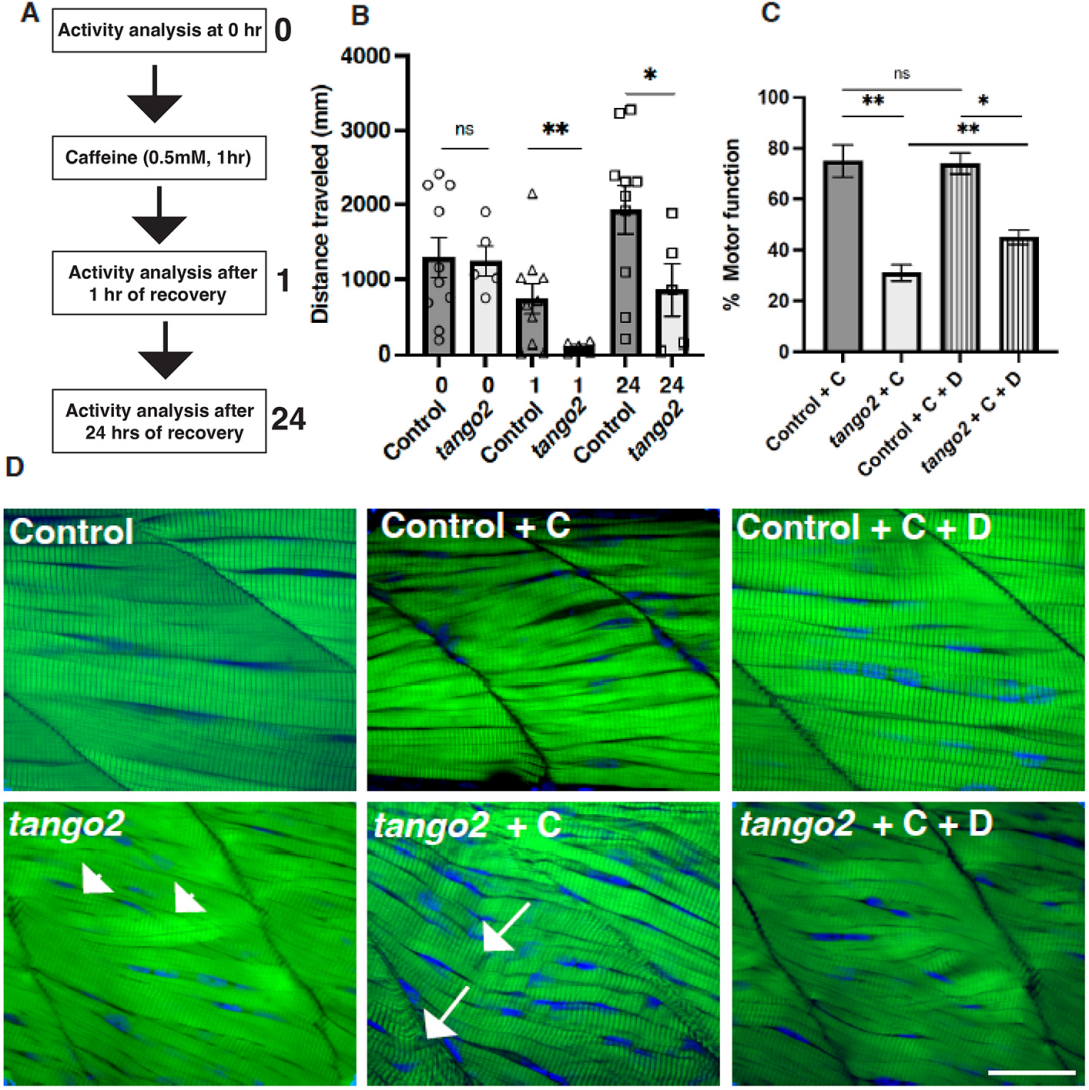
***tango2* mutants exhibit increased sensitivity to caffeine-induced SR stress that is partially reversed by dantrolene.** (A) Schematics for caffeine treatment. (B) Effect of caffeine treatment on total swimming distance by control and *tango2* mutants. *n*=96. (C) Effect of dantrolene (‘D’) treatment on motor function in caffeine (‘C’)-treated control and *tango2* mutants. Data were normalized to swimming distance before caffeine treatment within each group. *n*=48 in each group with 10-22 different genotypes. Data represent mean±s.e.m. Two-way ANOVA (mixed model) with multiple comparisons was performed between different groups. ns, not significant; *P<0.05, **P<0.01. (D) Whole-mount phalloidin staining (green) of control and *tango2* mutants exposed to caffeine and dantrolene. Arrowheads indicate misaligned myofibers and arrows indicate damaged myofibers. Nuclei are shown in blue. Images are representative of *n*=6 larvae in each group. Scale bar: 5 µm.

*tango2* mutant larvae exhibited normal motor function during early larval stages despite mild ultrastructural changes in a small group of myofibers (5-7 dpf, Fig. 4). This is similar to many TANGO2-deficient patients that exhibit normal motor function except for the development of acute muscle damage during rhabdomyolysis episodes. *tango2* mutant fish exhibited smaller or deflated terminal cisternae of the SR occupied by Ryr1 channels (Fig. 3). The SR is the regulator of excitation-contraction coupling in skeletal muscle through the release of Ca^2+^ by the Ryr1 channel and the reuptake of Ca^2+^ by the Serca channel (Lawal et al., 2020). Caffeine is an activator of Ryr1 that binds to Ryr1 and increases Ca^2+^ sensitivity. To test whether Tango2 deficiency increases susceptibility to muscle damage caused by SR stress, we treated control and *tango2* mutant larval fish (6 dpf) with caffeine. We quantified the swimming behavior by using an automated movement-tracking system. Acute caffeine exposure resulted in reduced swimming of control and *tango2* mutant fish. However, the effect was more severe in *tango2* mutants than in control siblings (Fig. 4A,B). Whereas control fish recovered completely after 24 h of caffeine exposure, *tango2* mutants failed to revert to the normal level of motor function.

Dantrolene is a Ryr1 antagonist and protects against hypersensitivity of Ca^2+^ release from the SR. Dantrolene is used clinically to control malignant hyperthermia and rhabdomyolysis. Therefore, we tested the effectiveness of dantrolene in improving acute muscle dysfunction in *tango2* mutants induced by caffeine exposure. Control and *tango2* mutant larval zebrafish (6 dpf) were treated with caffeine or caffeine and dantrolene, and motor function (normalized to pre-caffeine treatment) was analyzed. Caffeine treatment resulted in highly reduced motor function in *tango2* larval zebrafish compared to that in controls (Fig. 4C). Treatment with dantrolene resulted in a small and significant improvement in muscle function in *tango2* mutants. Whole-mount phalloidin staining of the myotome revealed a disorganized myotome in *tango2* mutants at the basal state, with several myofibers lacking the parallel myofiber organization seen in the controls (Fig. 4D, arrowheads). Although no significant myofiber breakdown or atrophy was observed upon caffeine exposure, *tango2* mutants showed disarray of the sarcomere banding pattern, with the widening of A-bands between adjacent I-bands (area between phalloidin-stained sarcomeres) (Fig. 4D, arrows), which were rescued with dantrolene treatment. Although improved motor function and skeletal muscle structure were observed in *tango2* mutants, dantrolene treatment did not result in a complete rescue of muscle structure and function to normal levels.

### Exercise-induced skeletal muscle damage in Tango2 deficiency

**Fig. 5.**
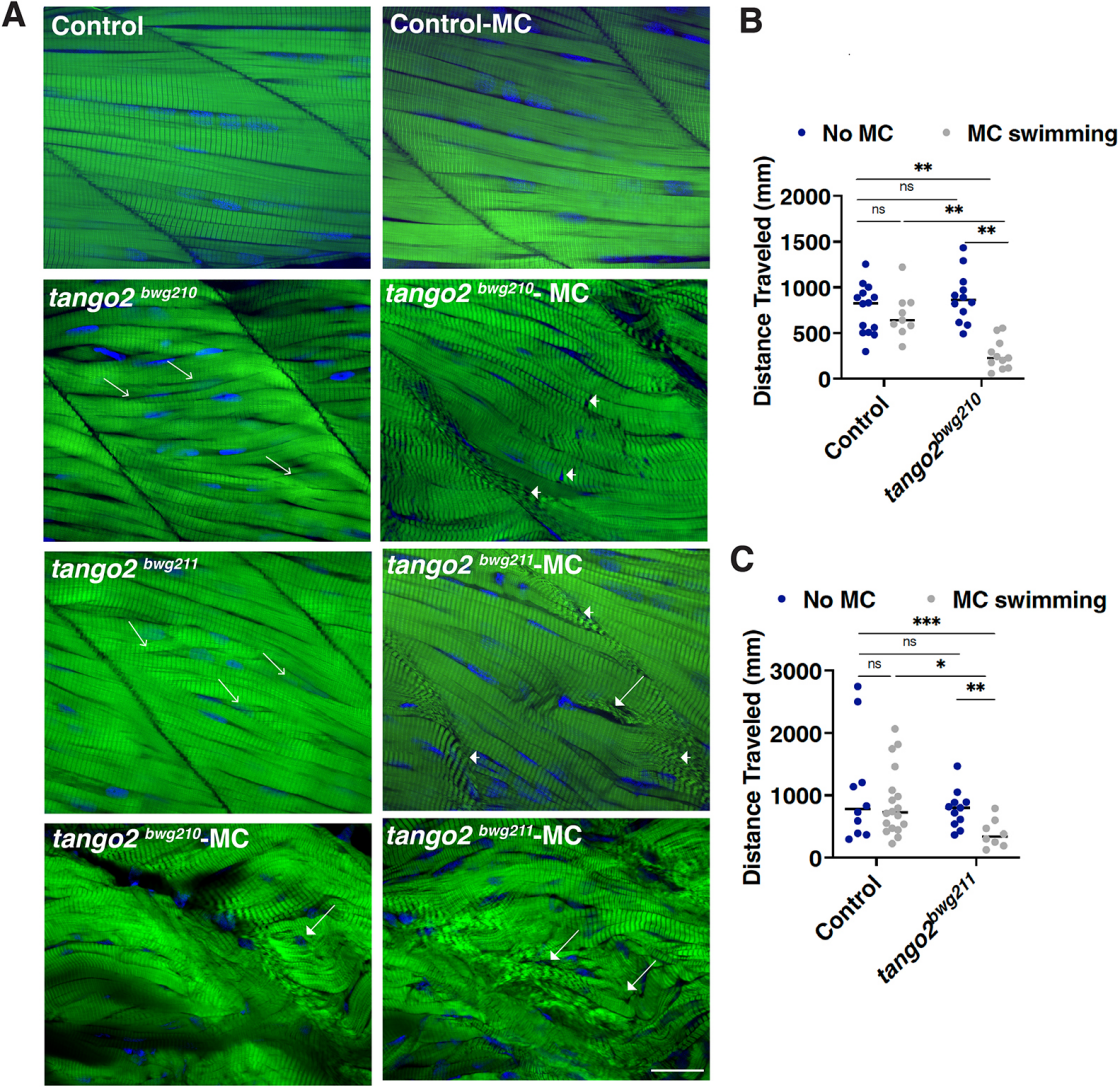
**Exercise-induced skeletal muscle damage in Tango2 deficiency.** (A) Whole-mount (green) phalloidin staining of control and *tango2* mutants (*tango2*^bwg210^ and *tango2*^bwg211^) in the presence or absence of methylcellulose (MC) treatment. *tango2* mutants exhibited extensive overstretched sarcomeres (arrows) and damaged myofibers (arrowheads) on swimming in the MC-containing water compared to those in controls (*n*=6-8 in each group). Nuclei are shown in blue. Images are representative of *n*=6 larvae in each group. Scale bar: 5 µm. (B,C) Quantification of the swimming behavior of control and *tango2* mutants (*tango2*^bwg210^ and *tango2*^bwg211^) before and after MC treatment (*n*=96). Two-way ANOVA (mixed model) with multiple comparisons was performed between different groups. ns, not significant; *P<0.05; **P<0.01; ***P<0.005.

TANGO2 deficiency in patients is associated with rhabdomyolysis. However, the stress conditions leading to rhabdomyolysis in these patients remain mostly unknown. Exercise-induced rhabdomyolysis is the most common trigger of muscle damage in susceptible individuals with other genetic forms of rhabdomyolysis. Mechanical loading of the skeletal muscle in control and *tango2* mutants (8 dpf) was induced by increasing the viscosity of the swimming water with the inert polymer methylcellulose. Subsequently, the effect of mechanical loading on skeletal muscle structure and function was analyzed in two different alleles of *tango2*: *tango2*^bwg210^ and *tango2*^bwg211^. Whole-mount phalloidin staining of the myotome revealed that control larval myofibers were organized in a parallel manner. *tango2* mutant myofibers appeared to be less structured, with many myofibers in both mutants lacking the parallel organization observed in the control muscle (Fig. 5A, open arrows). Quantification of the swimming behavior showed no significant differences between the spontaneous swimming behavior of control and *tango2* mutant alleles (Fig. 5B,C). Swimming in the methylcellulose-containing water resulted in extensive over-stretched and misaligned sarcomeres, with widened A-bands in the *tango2* mutant myofibers (Fig. 5A, arrowheads). Several of these mutants (3-5%) also exhibited extensive myofiber disorganization with hypercontracted broken myofibers after swimming in 1% methylcellulose, indicating muscle wasting (Fig. 5A, solid arrows). Quantification of spontaneous movement showed a significant decrease in the swimming capacity of *tango2* mutants after swimming in methylcellulose-containing water, but no differences were observed for control larval fish (Fig. 5B,C). Therefore, Tango2 deficiency increases susceptibility to exercise-induced skeletal muscle damage and impaired motor function.

### Altered lipid profiles in Tango2 deficiency

**Fig. 6.**
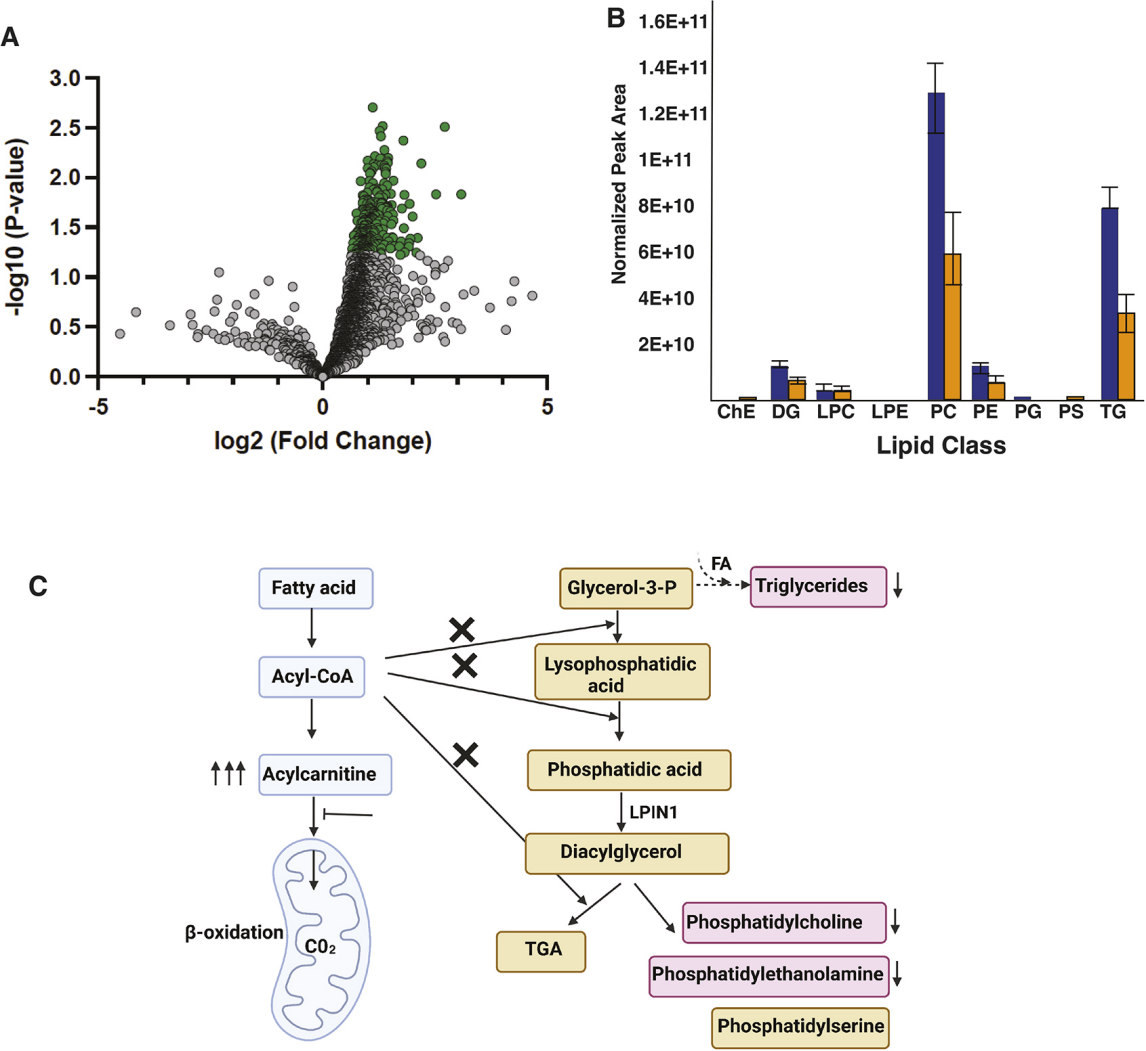
**Changes in lipid composition in Tango2 deficiency.** (A) Volcano plot of lipid log_2_(fold change) values in controls versus *tango2* mutants (4 weeks). Fold differences are calculated as the ratio of lipids in WT to those in *tango2* mutants. Dots in green represent the lipids with the lowest levels in *tango2* mutants in comparison to controls (*P*<0.05). (B) Normalized profiles of different lipids classes (blue, control; orange, *tango2* mutants). ChE, cholesterol ester; DG, diglyceride; LPC, lysophosphatidylcholine; LPE, lysophosphatidylethanolamine; PC, phosphatidylcholine; PE, phosphatidylethanolamine; PG, phosphatidylglycerol; PS, phosphatidylserine; TG, triglyceride. Data show the mean±s.e.m. Unpaired two-tailed *t*-test was used to determine significance. *P<0.05. (C) Working model of the effect of Tango2 deficiency on lipid metabolism: *tango2* mutants exhibit a decreased abundance of phospholipids and triglycerides (pink) indicating a defect in the glycerol-3-P pathway. The protein product of *LPIN1*, a rhabdomyolysis-causing gene, catalyzes diacylglycerol synthesis in the glycerol-3-P pathway. Triglycerides are energy stores synthesized using glycerol-3-phosphate and fatty acids (FA) and are reduced in Tango2 deficiency. Some patients' fibroblasts exhibit acylcarnitine accumulation, which may limit acyl-CoA availability for the glycerol-3-P pathway, resulting in phospholipid deficiency. TGA: Triacylglycerol.

Most cellular lipids are synthesized in the ER/SR, the central hub to regulate cellular lipid composition in response to intrinsic, homeostatic and environmental factors. Fibroblasts from TANGO2-deficient patients exhibit abnormal accumulation of fatty acids (Heiman et al., 2022). However, owing to clinical heterogeneity in patient samples, a clear correlation between lipids in disease pathology has not been established (Jennions et al., 2019). To comprehensively characterize the effect of Tango2 deficiency on the content and composition of structural lipids at the basal level, lipidomics was performed in control and *tango2* mutants (4 weeks of age) (Fig. 6A; Table S1). *tango2* mutants revealed a significant decrease in the abundance of phosphatidylcholine (PC), triglycerides and phosphatidylethanolamine (PE) (Fig. 6B; Table S2). The PC species that showed the most decrease in the mutants contained zero to seven double bonds among the fatty acyl tails [e.g. PC (15:0_22:6), PC (16:1_22:6), PC (17:0_20:3), PC (32:6) and PC (38:7)]. Analysis of the individual tails revealed that each of these lipids contained saturated, saturated and unsaturated, or unsaturated fatty acids, ranging in size from C15:0 to C39:2. PC is metabolized to lysophosphatidylcholine (LPC) and free fatty acids (Table S1). Reduced levels of LPC were also observed in *tango2* mutants, suggesting that a low abundance of PC subsequently results in decreased levels of downstream lipids such as LPC in Tango2 deficiency. PE with reduced abundance in mutants also contained saturated and unsaturated fatty acids with 17-22 carbon fatty acids (Table S1). Triglyceride levels were also significantly reduced in *tango2* mutants compared to those in controls. Triglycerides are stored as lipid droplets in skeletal muscle and can be hydrolyzed to produce fatty acids for energy production through β-oxidation and oxidative phosphorylation. The length of fatty acid chains in most triglycerides reduced in mutants contained 16-18 carbon atoms and saturated and unsaturated fatty acids (Table S1). No significant changes were seen in other lipid classes in Tango2 deficiency. Quantification of PC, which showed the highest reduction in *tango2* mutants at 4 weeks of age, showed a similar reduction in mutants at early larval stages (Fig. S3A). This suggests that most lipid defects observed in mutants are also present during early larval stages and are not a downstream secondary effect of disease progression. The ER/SR harbors enzymes for the glycerol-3-phosphate pathway for the synthesis of phospholipids, which are major building blocks for lipids in the cellular membrane. Quantification of the glycerol-3-phosphate pathway enzymes that catalyze lysophosphatidic acid to triacyl glycerol and phospholipids revealed a significant downregulation of *agpat2*, *lpin1* (*lpin1a*) and *dgat1a* in *tango2* mutants (Fig. S3B). Moreover, caffeine or mechanical loading further led to a decrease in gene expression for all the enzymes in the glycerolipid pathway, including *gpat3*, that exhibited normal levels without any extrinsic trigger. Therefore, the overall abundance of major membrane and cellular lipids synthesized through the ER/SR is significantly decreased in *tango2* mutant zebrafish.

## DISCUSSION

Rhabdomyolysis is a complex condition with several clinical complications and entails the rapid dissolution of damaged skeletal muscle, often leading to a life-threatening condition (Cabrera-Serrano and Ravenscroft, 2022). The rhabdomyolytic state is induced by a combination of environmental factors such as infections, fasting, drugs, medications, heat, and other unknown triggers and predisposing genotypes. However, a lack of a clear understanding of the intrinsic processes contributing to disease pathology precludes the assessment of different triggers and their interaction with genetic susceptibilities in rhabdomyolysis. To address these questions, we developed a knockout zebrafish model of Tango2 deficiency that recapitulates functional and pathological changes observed in TANGO2-deficient patients and provides the mechanism for skeletal muscle defects.

Our *tango2* mutant zebrafish showed normal embryonic development and motor function during larval stages. This is similar to TANGO2-deficient patients, who typically do not exhibit early embryonic developmental defects and develop metabolic crises and rhabdomyolysis during early or late childhood (Lalani et al., 2016). This similarity demonstrates that the *tango2* zebrafish is a valuable model for understanding the clinical onset and disease trajectories leading to serious clinical complications. Recently, another allele of zebrafish *tango2* was described that showed normal muscle function and survival of the mutants obtained from heterozygous parents owing to the presence of maternal *tango2* mRNA. Mutants obtained from mutant parents exhibited larval lethality and defects in muscle structure and function (Sun et al., 2022). The mutants obtained from heterozygous or mutant parents in our study showed similar survival, normal motor function and lack of maternal *tango2* mRNA (Fig. 1C,E; Fig. S1A,B) (Sun et al., 2022). As different sgRNAs were used to create fish lines in ours and the previous study, the differences in phenotypes between alleles could be due to some genetic background effect. Although the previous work showed the requirement of the *TANGO2* orthologue *HRG-9* in heme trafficking and overload in mitochondria in yeast and *Caenorhabditis elegans*, no defects in heme trafficking were observed in *tango2* mutant zebrafish. Heme metabolism is critical for skeletal muscle function (Alves de Souza et al., 2021). A lack of difference in heme synthesis in control and *tango2* mutant fish suggests that Tango2 is not required for *in vivo* heme metabolism in vertebrates under normal conditions. Despite defective mitochondria in a small number of myofibers, no significant differences in muscle function were observed in our *tango2* mutants in the absence of any extrinsic trigger.

Although *tango2* mutants exhibit normal swimming behavior, their skeletal muscle showed a disorganized myotome and a small number of abnormal mitochondria and SR. This suggests that Tango2 deficiency results in intrinsic defects in skeletal muscle structure and function; still, the basal threshold function of the skeletal muscle is sustained. However, under certain stress conditions, these defects may prevent skeletal muscle from functioning beyond a basal threshold or may result in muscle breakdown and other abnormalities. This is evident from caffeine exposure or mechanical loading of skeletal muscle, which reduced motor function and increased myofiber damage in *tango2* mutants compared to the effects in controls. Mechanical loading resulted in extensive sarcomere disorganization, with damaged and broken myofibers in the severely affected *tango2* mutants. Prolonged mechanical loading in normal skeletal muscle can result in overstretched sarcomeres, myofibril misalignment and myofiber atrophy. Upon rest, normal skeletal muscle undergoes rapid restructuring and recovery (Krippendorf and Riley, 1994; Newham et al., 1983). Unlike control larvae, *tango2* mutants failed to restore normal skeletal muscle structure. This is similar to the exertional rhabdomyolysis, commonly observed in rhabdomyolysis patients (Carneiro et al., 2021). Previous studies have shown that increased exercise results in direct membrane damage with Ca^2+^-handling dysfunction, leading to increased Ca^2+^ concentration and concordant muscle contractions increasing the energy deficit (Aldrich et al., 2021). Finally, this results in the activation of Ca^2+^-dependent proteases and phospholipases, which contributes to the damage to myofibrillar and cytoskeletal proteins (Scalco et al., 2015). A similar mechanism may be contributing to skeletal muscle damage in Tango2 deficiency, as shown by the increased sensitivity of *tango2* mutants to caffeine, which binds to Ryr1 channels in the SR and induces the release of Ca^2+^ in the cytosol (Chirasani et al., 2021).

To identify the basal intrinsic defects in Tango2 deficiency, we performed lipidomics in control and *tango2* mutants as TANGO2-deficient patient-derived cell lines show abnormal accumulation of fatty acids or acylcarnitines (Schymick et al., 2022; Bérat et al., 2021). However, these findings are quite divergent and failed to provide a clear outcome owing to wide clinical heterogeneity in patients' samples. Our lipidomic analysis identified a significant reduction in phospholipids and triglycerides in Tango2 deficiency. These phospholipids contained unsaturated and mono- or poly-saturated fatty acid chains, with decreased large-chain fatty acids (16C-38C). PC and PE are the most abundant phospholipids (50% of total lipids) and a decrease in these lipid species may increase the susceptibility to membrane damage by regulating membrane stability and fluidity. Previous studies have shown that a reduction in phospholipids results in skeletal muscle myopathy (Ferrara et al., 2021), and, therefore, decreased amounts of phospholipids in Tango2 deficiency could underlie the muscle weakness seen in TANGO2-deficient patients. Phospholipid synthesis occurs predominantly through the glycerol-3-phosphate pathway (Fig. 6C). LPIN1 catalyzes an essential step of this process, and mutations in *LPIN1* are the most common cause of severe recurrent rhabdomyolysis through loss of cell membrane integrity and myofiber dysfunction (Zeharia et al., 2008). Many glycerol-3-phosphate pathway enzymes, including *lpin1*, were downregulated in Tango2 deficiency. This suggests that a decrease in glycerol-3-phosphate pathway enzymes associated with reduced phospholipid levels may further increase the susceptibility to myofiber damage exacerbated under extrinsic stress. Glycerol-3 phosphate is a substrate for triglyceride synthesis for energy storage (Wu et al., 2015). Reduced levels of triglycerides in *tango2* fish further point to defects in glycerolipid homeostasis in Tango2 deficiency. Some TANGO2-deficient patients also exhibit acylcarnitine accumulation (Schymick et al., 2022), suggesting that defects in the glycerol-3-phosphate pathway in Tango2 deficiency may prevent utilization of acyl-CoA, thus leading to acylcarnitine accumulation (Fig. 6C). Acylcarnitine is normally metabolized by the β-oxidation pathway. As mitochondrial defects are also observed in Tango2 deficiency (Figs 2 and 3), decreased metabolism of acylcarnitines may lead to their accumulation, which is toxic for several organs, including the skeletal muscle, heart and liver. We did not observe any significant changes in the acylcarnitines in Tango deficiency. As lipidomics analysis in *tango2* mutants was performed in the basal state, the accumulation of acylcarnitines seen in some TANGO2-deficient patients could be triggered by metabolic or other stress states.

Although no functional defects were observed at the basal state during early larval stages in *tango2* mutants, a variability in survival rate was observed during larval and juvenile stages. During the first few days of development, zebrafish embryos and larvae survive on nutrients provided by the egg yolk. However, as these animals transition to external feeding at 5-6 dpf, different amounts of nutrients and swimming (exercise) may elicit variable phenotypes in affected mutants. Similarly, human TANGO2-deficient patients have variable rhabdomyolysis onset, which may be caused by differences in behavior or metabolic processes. This is further evident from recent studies that showed that nutrient stress controls lipid homeostasis in *TANGO2* disease pathogenesis through the regulation of acyl-CoA by phosphatidic acid (Lujan et al., 2023). In another recent study, treatment with vitamin B5, a coenzyme A precursor, rescues seizures in a *Drosophila* model of tango2 deficiency (Asadi et al., 2023). This suggests that Tango2 deficiency results in cellular and membrane lipid defects in the basal stage, which are exacerbated by extrinsic signals such as nutrient stress, caffeine and exercise and are potential risk factors for the development of metabolic crisis and rhabdomyolysis in TANGO2-deficient patients. Future studies on how Tango2 regulates these processes will further improve our understanding of *TANGO2*-related disorders.

## MATERIALS AND METHODS

### Zebrafish lines

Fish were bred and maintained using standard methods as described (Westerfield, 2000). All procedures were approved by the Brigham and Women's Hospital Animal Care and Use Committee. *tango2*^bwg210^ and *tango2*^bwg211^ zebrafish lines were created in our laboratory by the CRISPR-Cas9 approach. Zebrafish embryonic (0-2 dpf), larval (3-45 dpf), juvenile (45 dpf-3 months) and adult (3 months) stages were defined as described previously (Kimmel et al., 1995). Zebrafish clutches exhibiting >10% lethality (0-1 dpf) were excluded from the study. All studies presented in this work were performed on *tango2*^bwg211^ mutants obtained from heterozygous parents, unless otherwise specified.

### Genotyping assays for *tango2* lines

DNA was extracted from zebrafish larvae or fin clips of adult zebrafish, genotyped by PCR, and analyzed by a 2% agarose gel (Bennett et al., 2018). The PCR primer sequences used for genotyping were: 5′-TGGGAATTAGCAAACGAGGA-3′ and 5′-ATGGCTGAAAGAGCTGTGCT-3′.

### Real time PCR and cDNA sequencing in controls and mutant *tango2*

cDNA was analyzed by gel and Sanger sequencing using heterozygous siblings as controls to detect the presence of maternal mRNA in wild-type and mutant siblings. Total RNA was isolated from individual wild-type, mutant and heterozygous siblings obtained from *tango2^bw211^* heterozygous parents (1 month of age) using the RNeasy Fibrous Tissue Kit (QIAGEN, 74705) according to the manufacturer's instructions. cDNAs were synthesized from 500 ng of total RNA using the SuperScript III First-Strand Synthesis System (Thermo Fisher Scientific, 18080051) and random hexamers. The primers used for PCR and Sanger sequencing were: forward primer, 5′-TCCAAAGCTGCGGAATTCT-3′, and reverse primer, 5′-CTGTGAGGAGATTGAAGCCATT-3′. Quantitative real-time PCR was performed using the SYBR green assay as described previously (Bennett et al., 2018).

### Western blotting

Zebrafish larvae at 30 dpf were homogenized in buffer containing 20 mM Tris-Cl (pH 7.6), 50 mM NaCl, 1 mM EDTA, 0.1% NP-40 and complete protease inhibitor cocktail (Roche Applied Sciences, Indianapolis, IN, USA). Following centrifugation at 11,000 ***g*** at 4°C for 15 min, the protein concentration in supernatants was determined by BCA protein assay (Pierce, Rockford, IL, USA). Proteins were separated by electrophoresis on 4-12% gradient Tris-glycine gels (Invitrogen) and transferred onto polyvinylidene difluoride membranes using an iBlot dry blotting system (Thermo Fisher Scientific). Membranes were blocked in PBS containing 5% casein and 0.1% Tween 20 and incubated with rabbit polyclonal anti-Tango2 (1:250, 27846-1-AP, Proteintech), mouse monoclonal anti-tubulin (1:500, T9026-100UL, Sigma-Aldrich) or anti-GAPDH (1:2000, 2118S, Cell Signaling Technology) primary antibodies. After washing, membranes were incubated with horseradish peroxidase-conjugated anti-rabbit (1:2500, 170-6515) or anti-mouse (1:5000, 170-6516) IgG secondary antibodies (Bio-Rad, Hercules, CA, USA). Proteins were detected using the SuperSignal chemiluminescent substrate kit (Pierce).

### Myofiber isolation and immunofluorescence

Myofibers were isolated from control or *tango2* larval zebrafish (45 dpf), as described previously with minor modifications (Ganassi et al., 2021). Skinned zebrafish muscle samples were treated with collagenase for 90 min and triturated to release the myofibers. Myofibers were centrifuged at 1000 ***g*** for 60 s, washed, and resuspended in Dulbecco's modified Eagle medium (DMEM, Thermo Fisher Scientific). Myofibers were plated on laminin-coated eight-chamber Permanox slides (Thermo Fisher Scientific) for further analysis. Fixed cells were blocked in 10% goat serum and 0.3% Triton X-100, incubated in the primary antibody overnight at 4°C, washed in PBS, incubated in the secondary antibody for 1 h at room temperature, washed in PBS, then mounted with Vectashield Mounting Medium (Vector Laboratories, Burlingame, CA, USA). The primary antibodies used were anti-Tango2 (1:250, 27846-1-AP, Proteintech), mouse monoclonal anti-sarcomeric α-actinin (1:100, A7732, Millipore Sigma), mouse monoclonal anti-Ryr1 (1:100, R129-100UL, Millipore Sigma) and anti-Tomm20 (1:100, MABT166, Millipore Sigma). Alexa Fluor 568-phalloidin (1:100, Thermo Fisher Scientific, A12380) was used to label F-actin. After washing in PBS several times, samples were incubated with anti-mouse Alexa Fluor 594 (1:100, A-11005) secondary antibody (Thermo Fisher Scientific). Imaging was performed using a Nikon Ti2 spinning disk confocal microscope and colocalization analysis was performed on *z*-stack projections.

### Caffeine and dantrolene treatment

Zebrafish (6 dpf) obtained from heterozygous matings were placed in individual wells of a 48-well dish and swimming behavior was analyzed at the basal level. Caffeine (Millipore Sigma, C0750) and dantrolene (Millipore Sigma, D9175) treatments were performed as previously described with some modification (Endo et al., 2022). E3 water was replaced with 0.5 μM caffeine-containing E3 water, and fish were incubated for 1 h. Subsequently, caffeine was replaced with normal E3 water. Swimming behavior was analyzed again after 1 h of recovery and after 24 h of recovery by the automated tracking system. For dantrolene treatment, zebrafish were incubated with 5 μM dantrolene for 2 h, followed by 1 h incubation with caffeine and dantrolene. Swimming behavior was analyzed before the treatment, after 1 h of recovery, and after 24 h of recovery by the automated tracking system.

### Methylcellulose assay and whole-mount phalloidin staining

Zebrafish larvae (7 dpf) obtained from *tango2* heterozygous matings were individually placed in 48-well dishes in E3 water, and swimming behavior was quantified using the Zantiks MWP automated tracking system (Zantiks, Cambridge, UK). Subsequently, E3 water was replaced with 1% methylcellulose-containing E3 water for 7 h at 28.5°C. Zebrafish larvae were washed two times with the E3 water, and swimming behavior was quantified again. Larval heads were collected for genotyping, and bodies were fixed in 4% paraformaldehyde. Whole-mount phalloidin staining was performed as previously described (Casey et al., 2023).

### PC quantification assay

PC quantification was performed on 8 and 30 dpf control and *tango2* mutants using the PC assay kit (MAK040, Millipore Sigma) according to the manufacturer's instructions. Briefly, 30 pooled control or mutant larval fish (7 dpf) or individual fish (30 dpf) were homogenized in PC assay buffer, and the supernatant was collected after centrifugation. PC hydrolysis enzyme, PC development mix and fluorescent peroxidase substrate were added to the tissue extracts in PC buffer and incubated for 30 min at room temperature. The fluorescence intensity was measured (λ_excitation_=535 nm, λ_emission_=587 nm). Data were normalized with the total body weight of each sample.

### Lipidomic profiling

Control and *tango2* mutant zebrafish (4 weeks, *n*=5 each) were homogenized with 1 ml of methyl-tert-butyl ether (MBTE; Thermo Fisher Scientific). 300 μl of methanol with an internal standard was added, and samples were mixed for 10 min. 200 μl of water was added to facilitate phase separation. The extracts were centrifuged at 14,000 ***g*** for 10 min. The top layer was removed, dried and reconstituted in 150 μl of isopropyl alcohol (IPA) for analysis. Analysis was performed using a Q Exactive Plus mass spectrometer (Thermo Fisher Scientific) coupled to a Waters Acquity H-Class liquid chromatography system (Waters). A 100×2.1 mm, 2.1 µm BEH C18 column (Waters) was used for separations. The following mobile phases were used: phase A, 60% acetonitrile (ACN)/40% H_2_O; phase B, 90% IPA/10% ACN; both mobile phases contained 10 mM ammonium formate and 0.1% formic acid. A flow rate of 0.2 ml/min was used. The starting composition was 32% phase B, which increased to 40% phase B at 1 min (held until 1.5 min), then 45% phase B at 4 min. This was increased to 50% phase B at 5 min, 60% phase B at 8 min, 70% phase B at 11 min, and 80% phase B at 14 min (held until 16 min). At 16 min, the composition switched back to starting conditions (32% phase B) and was held for 4 min to re-equilibrate the column. Samples were analyzed in positive/negative-switching ionization mode with top-five data-dependent fragmentation.

Raw data were analyzed by LipidSearch (Thermo Fisher Scientific). Lipids were identified by MS2 fragmentation (mass error of precursor=5 ppm, mass error of product=8 ppm). The identifications were generated individually for each sample and then aligned by grouping the samples [oxidized 1-palmitoyl-2-arachidonoyl-sn-glycero-3-phosphocholine (OxPAPC; 870604, Avanti Lipids) was used to detect and monitor oxidized lipids in control and *tango2* mutant zebrafish]. Normalization was performed using EquiSplash from Avanti Polar Lipids using internal standards and the body weight of individual samples. Samples were normalized and biological replicates were averaged. *P*-values and fold change values were calculated as instructed and as previously described (Aguilan et al., 2020). A *P*-value less than 0.05 was considered to be statistically significant.

### Zebrafish locomotion assay

Zebrafish swimming behavior was quantified using the Zantiks MWP automated tracking system. Larval zebrafish (5-7 dpf) were placed individually by randomization into each well of a 48-well plate, and their swimming behavior was recorded for 50 min (10 min light, 10 min dark, 10 min light, 10 min dark, 10 min light, end). Four independent anonymized trials were performed, and the total distance and cumulative duration of the movement were recorded.

### Electron microscopy

Zebrafish embryos (8 dpf) were used to perform transmission electron microscopy. Heads of individual larval fish were removed for genotyping, and bodies were fixed in formaldehyde–glutaraldehyde–picric acid in cacodylate buffer overnight at 4°C, followed by osmication and uranyl acetate staining. Subsequently, embryos were dehydrated in a series of ethanol washes and embedded in TAAB Epon (Marivac, Halifax, NS, Canada). Sections (95 nm) were cut with a Leica UltraCut microtome, picked up on 100 mm Formvar-coated copper grids and stained with 0.2% lead citrate. Sections were viewed and imaged using a JEOL 1200EX transmission electron microscope at the Harvard Medical School Electron Microscopy Core.

### Quantification and statistical analysis

All samples were anonymized till final analyses, and statistical analyses were performed using GraphPad Prism 9.

## Supplementary Material

10.1242/dmm.050092_sup1 Supplementary information
