# Supplementary material for: Intrinsic and extrinsic regulation of rhabdomyolysis susceptibility by Tango2
Source: Dis Model Mech. 2023 Sep 5;16(9):dmm050092. doi: 10.1242/dmm.050092 (PMC10499024; doi:10.1242/dmm.050092)
Supplement: Supplementary information [file dmm-16-050092-s1.pdf]

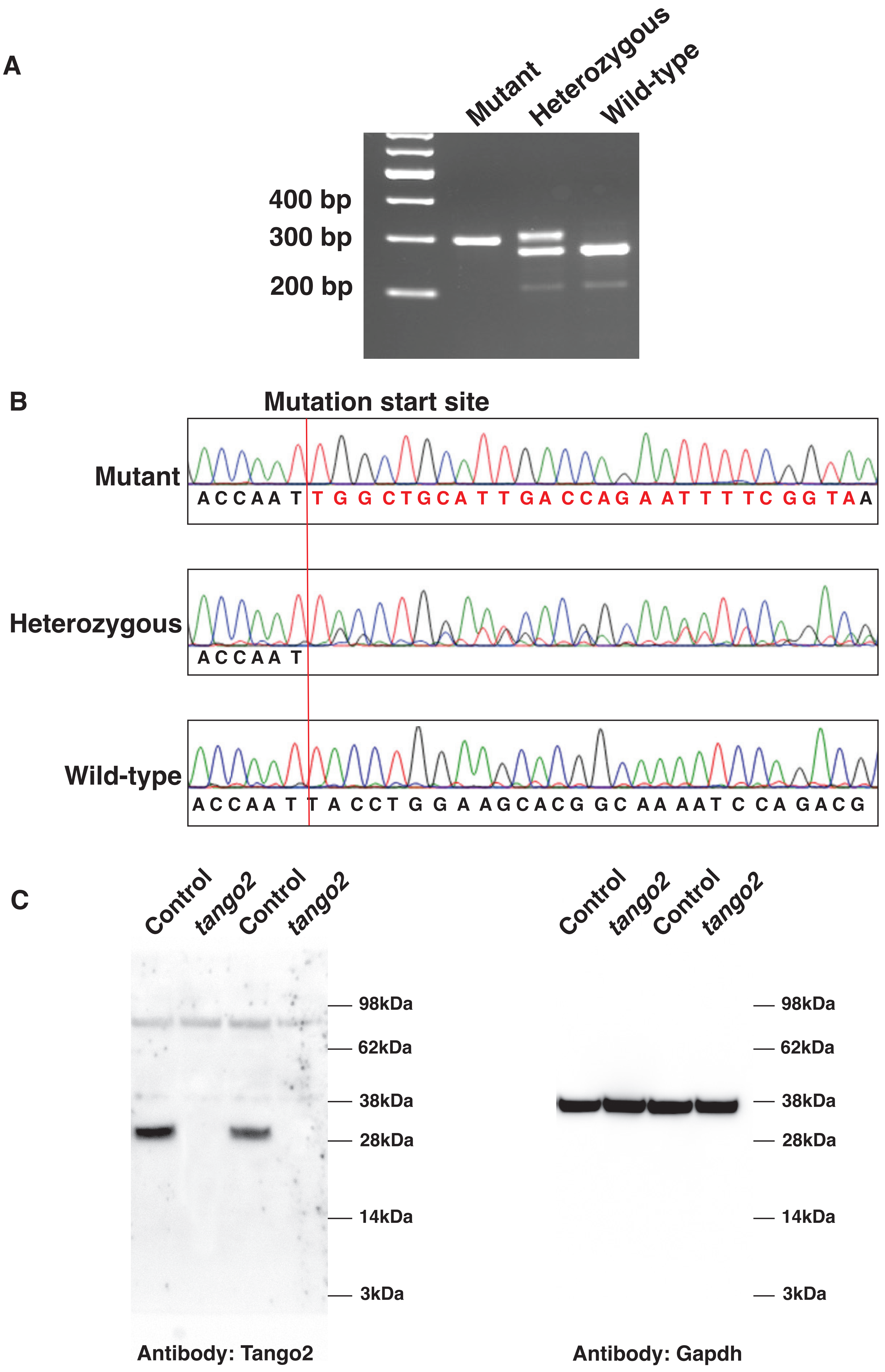

Disease Models & Mechanisms • Supplementary information

Fig. S1. ***tango2*** **mRNA analysis in controls and mutant** $\textbf{\textit{tango2}}^{\textbf{\textit{bwh21}}}$ **(c.226_227ins26) zebrafish siblings obtained from heterozygous parents.** (A) cDNA analysis from wild-type, heterozygous and mutant zebrafish on the gel (3%) demonstrated that mutant mRNA is stable (1 month age). (B) Sanger sequencing for controls and *tango2* mutant allele depicting lack of maternal wild-type transcript in the mutant zebrafish. (C) Raw western blots for Tango2 and Gapdh in control and *tango2* mutant zebrafish. Protein extracts were prepared from individual control or *tango2* mutant zebrafish at 30 dpf.

Disease Models & Mechanisms • Supplementary information

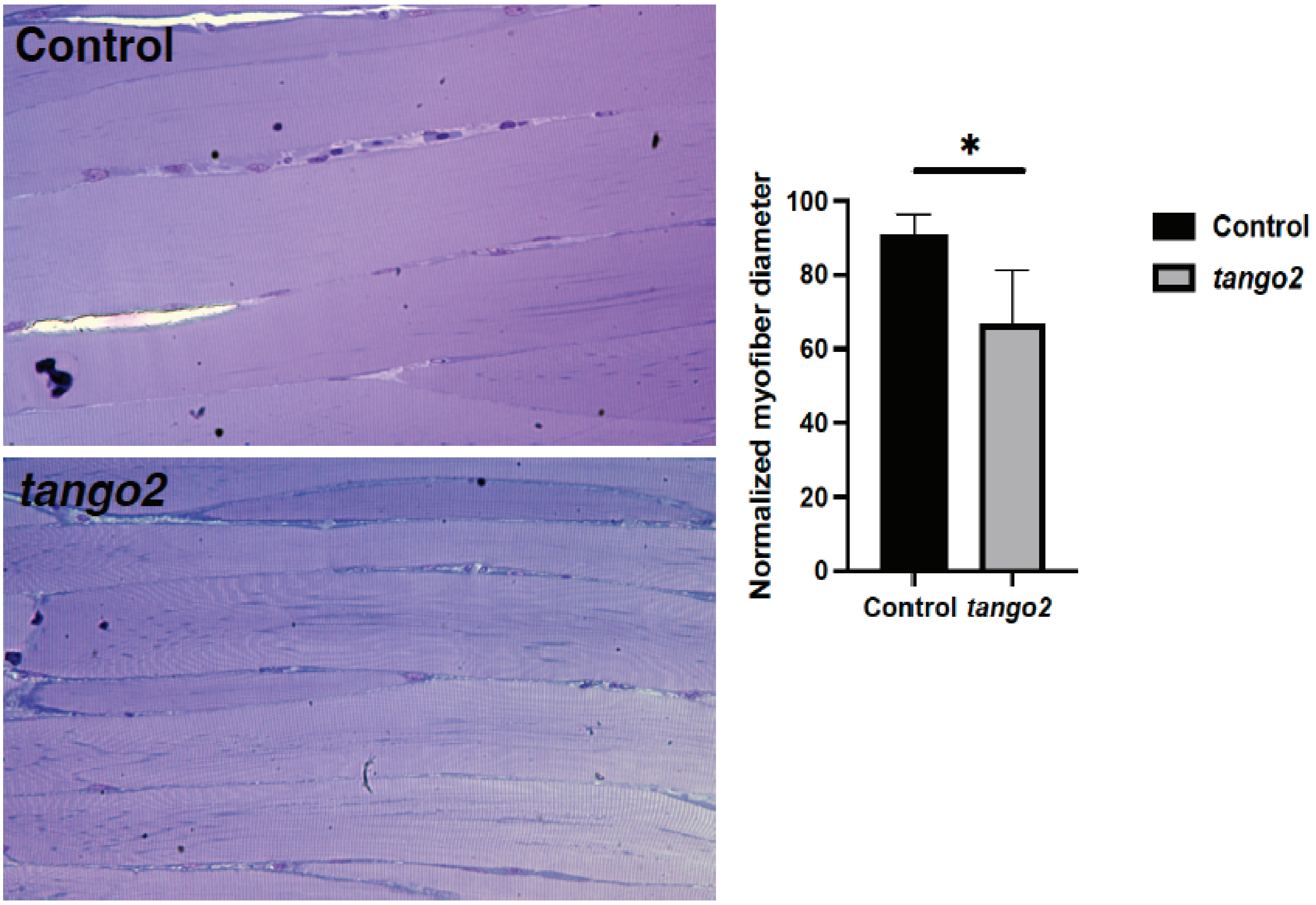

Fig. S2. *tango2* mutants exhibit reduced myofiber growth during the late larval stage. Histology of longitudinal skeletal muscle sections in control and tango2 mutants stained with toluidine blue (45 dpf). Quantification of the myofiber diameter showed reduced myofiber size in *tango2* mutants. n= 3 zebrafish in each group and data are presented as mean±S.E.M (unpaired t-test, parametric); *p<0.05.

Disease Models & Mechanisms • Supplementary information

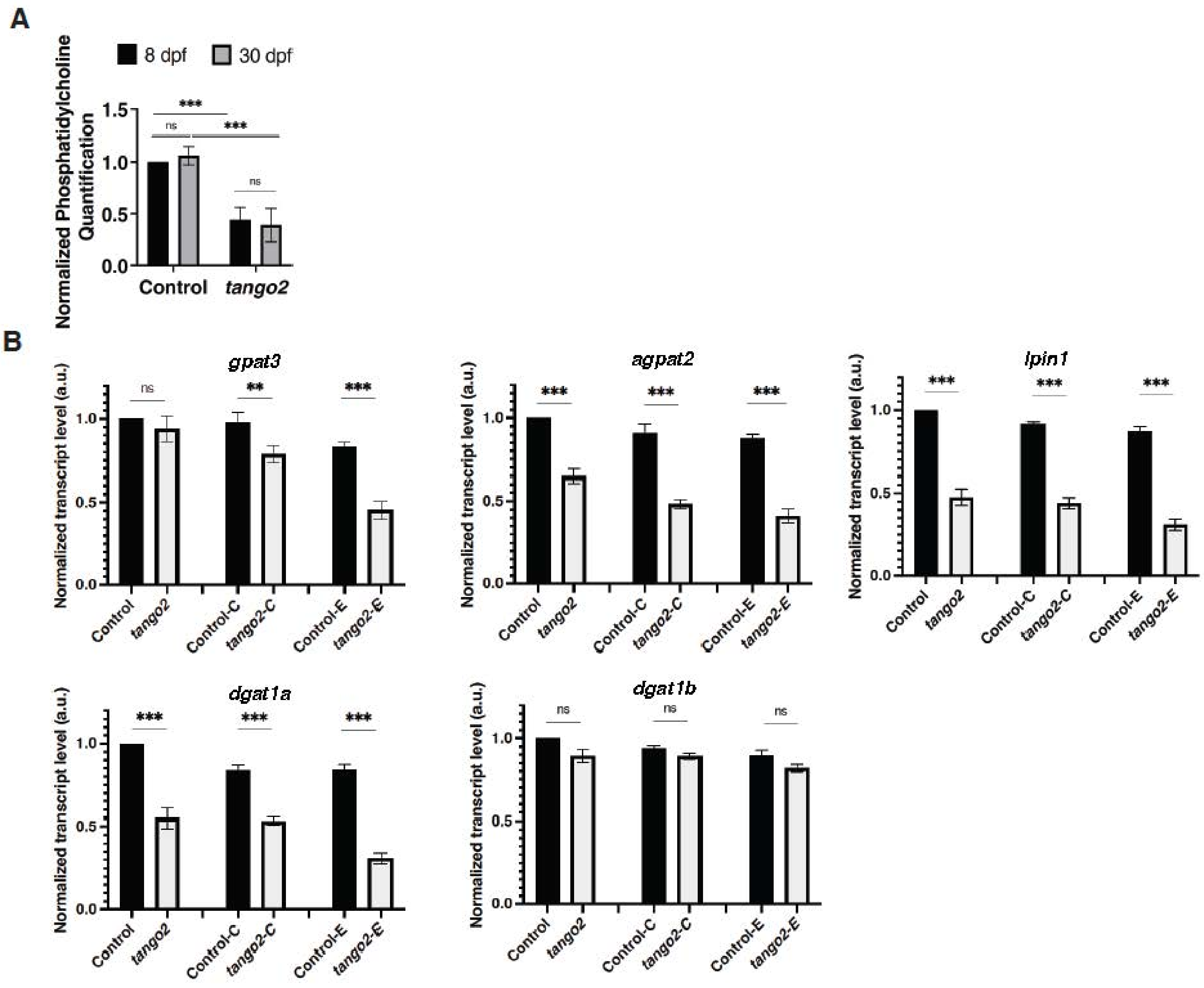
A
8 dpf
30 dpf
Normalized Phosphatidylcholine Quantification
1.5
1.0
0.5
0.0
***
ns
***
ns
Control
tango2
B
gpat3
agpat2
lpin1
dgat1a
dgat1b
Normalized transcript level (a.u.)
ns
**
***
Control
tango2
Control-C
tango2-C
Control-E
tango2-E

Fig. S3. Defects in glycerolipid pathway in Tango2 deficiency. (A) Quantification of phosphatidylcholine at 8 and 30 dpf in control and *tango2* mutants. (B) Quantification of transcripts of ER-localized glycerol-3-Phosphate pathway enzymes in the basal state, on caffeine exposure (C) or exercise by mechanical loading (E) at 8 dpf. n=10-12 in each group and data are presented as mean±S.E.M (unpaired t-test, parametric); (Non-significant: ns, *p<0.05, **p<0.01, ***p<0.005).

Table S1. Normalized global lipid profiles in control and *tango2* mutants (1 month age)

Click here to download Table S1

Table S2. Normalized lipid classes in control and *tango2* mutants (1 month age)

Click here to download Table S2

Disease Models & Mechanisms • Supplementary information
